# Retracted: Apoptosis and Molecular Targeting Therapy in Cancer

**DOI:** 10.1155/2020/2451249

**Published:** 2020-08-28

**Authors:** BioMed Research International

*BioMed Research International* has retracted the article titled “Apoptosis and Molecular Targeting Therapy in Cancer” [1]. The article was found to contain a substantial amount of material from previously published articles, including the following sources:

(i) John C Reed. “Apoptosis-targeted therapies for cancer”, Cancer Cell, 2003. 10.1016/S1535-6108(02)00241-6. [2] (Not Cited)

(ii) DB Longley, PG Johnston. “Molecular mechanisms of drug resistance”, The Journal of Pathology, 2005. 10.1002/path.1706. [3] (Not Cited)

(iii) Suparna Mazumder, Dragos Plesca and Alexandru Almasan. “A Jekyll and Hyde Role of Cyclin E in the Genotoxic Stress Response: Switching from Cell Cycle Control to Apoptosis Regulation”, Cell Cycle, 06/15/2007. 10.4161/cc.6.12.4432. [4] (Not Cited)

(iv) Plati, Jessica, Octavian Bucur, and Roya Khosravi-Far. “Apoptotic cell signaling in cancer progression and therapy”, Integrative Biology, 2011. DOI: 10.1039/C0IB00144A. [5] (Not Cited)

(v) C. Gullo, M. Au, G. Feng, and G. Teoh, “The biology of Ku and its potential oncogenic role in cancer,” Biochimica et Biophysica Acta—Reviews on Cancer, vol. 1765, no. 2, pp. 223–234, 2006. 10.1016/j.bbcan.2006.01.001. [6] (Cited as reference 222)
